# Obesity Paradox and the Effect of NT‐proBNP on All‐Cause and Cause‐Specific Mortality

**DOI:** 10.1002/clc.70044

**Published:** 2024-11-08

**Authors:** Rupinder Kaur Bahniwal, Nargiza Sadr, Colleen Schinderle, Cynthia J. Avila, Julie Sill, Rehan Qayyum

**Affiliations:** ^1^ Department of Medicine Eastern Virginia Medical School Norfolk Virginia USA; ^2^ Massey Comprehensive Cancer Center Virginia Commonwealth University Richmond Virginia USA

**Keywords:** healthy population, mortality, NT‐proBNP, obesity paradox

## Abstract

**Background:**

In heart failure patients, obesity is associated with better outcomes as compared to normal weight, a phenomenon called the obesity paradox.

**Objective:**

To examine if obesity modifies the relationship between NT‐proBNP and all‐cause and cause‐specific mortality in adults without coronary artery disease or heart failure history.

**Methods:**

We used the National Health and Nutrition Examination Survey (NHANES) data from 1999 to 2004 and linked it with mortality through December 31, 2019. Participants > 18 years were categorized into normal weight (BMI ≤ 25 kg/m^2^), overweight (BMI > 25–29.9 kg/m^2^) and obese (BMI > 29.9 kg/m^2^). NT‐proBNP levels were categorized as low (< 126 pg/mL) or high (≥ 126 pg/mL). Using Cox proportional hazard models, we examined effect modification by obesity using interaction, without and with adjusting for potential confounders.

**Results:**

Of the 12 621 participants, 2794 (22%) died during 202 859 person‐years follow‐up. In adjusted models, normal‐weight participants with high NT‐proBNP had 2 times higher all‐cause mortality risk than those with low NT‐proBNP (HR = 2.05; 95%CI = 1.74, 2.41; *p* < 0.001); however, this mortality risk was 27% lower in obese participants (HR interaction = 0.73; 95%CI = 0.59, 0.92; *p* = 0.008). Similarly, in normal‐weight participants, the difference in other‐cause mortality risk between high and low NT‐proBNP participants was significant in adjusted models (HR = 2.27; 95%CI = 1.81, 2.85; *p* < 0.001) and obese participants had 48% lower other‐cause mortality risk between those with high and low NT‐proBNP (interaction HR = 0.52; 95%CI = 0.36, 0.77; *p* = 0.001). Conversely, obesity did not modify the relationship between NT‐proBNP and cardiovascular or cancer mortality.

**Conclusions:**

In patients free of heart failure or coronary artery disease, obesity may be protective against mortality associated with high NT‐proBNP.

## Graphical abstract

Obesity, a recognized risk factor for cardiovascular disease (CVD), presents a paradoxical relationship with cardiovascular mortality but its association with other cause‐specific mortality is unknown. The study was conducted among 12 621 participants of NHANES (from 1999 to 2004) free of clinical coronary artery disease or heart failure. We found that elevated NT‐proBNP levels ( ≥ 126 pg/mL) were associated with greater all‐cause mortality, cardiovascular, cancer, and other‐cause mortality in individuals. Obese individuals with elevated NT‐proBNP ( ≥ 126 pg/mL), exhibited significantly lower all‐cause and other‐cause mortality rates compared to normal‐weight individuals with elevated NT‐proBNP. While elevated NT‐proBNP levels were associated with increased all‐cause and cause‐specific mortality, obesity did not alter the relationship between elevated NT‐proBNP and cardiovascular or cancer mortality. The observed associations remained significant even when accounting for potential confounders or competing causes of mortality.

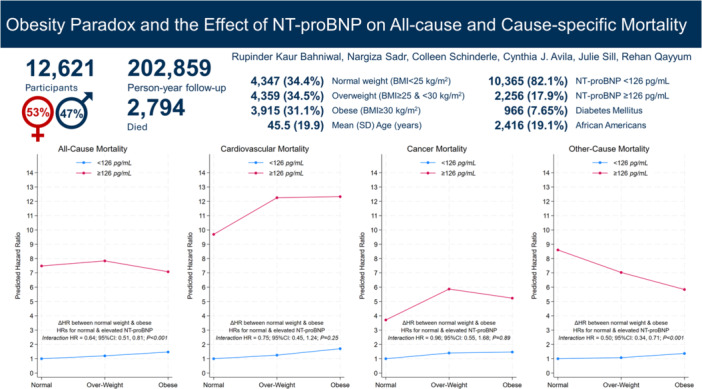

## 1 Introduction

Obesity, a recognized risk factor for cardiovascular disease (CVD), presents a paradoxical relationship with mortality. Referred to as the “obesity paradox,” this phenomenon is an effect modification by obesity on the association between CVD and mortality, whereby obese individuals with established CVD exhibit lower mortality rates as compared to normal‐weight individuals [1, 2]. This paradox appears specific to CVD mortality and has not been consistently observed in non‐CVD conditions, providing a need to better understand the nuanced relationship between obesity, CVD, and mortality [3, 4].

N‐terminal pro‐B‐type natriuretic peptide (NT‐proBNP) serves as a crucial biomarker for CVD, particularly heart failure. Its high sensitivity and specificity, coupled with a stable serum concentration, make it a preferred choice for diagnosis and risk assessment in acute and outpatient settings [5, 6, 7, 8]. In populations free of CVD, elevated NT‐proBNP and its active form, BNP, consistently demonstrate a robust association with mortality [9, 10, 11, 12]. These markers not only predict elevated mortality risk across diverse clinical settings and patient populations but also remain a potent predictor of death after adjusting for traditional risk factors and echocardiographic abnormalities [10]. By providing independent prognostic information beyond established clinical markers, NT‐proBNP's prognostic value transcends the presence or absence of CVD, thus offering a universal metric for mortality risk stratification [11].

While the association of NT‐proBNP with mortality in individuals free of CVD is well‐established, it is unknown whether obesity modifies this relationship. To address this knowledge gap, we investigated the obesity paradox in a population free of CVD and examined whether obesity modifies the relationship between NT‐proBNP and all‐cause and cause‐specific mortality. By disentangling this complex interplay in individuals free of CVD, we aimed to gain valuable clinical insights into the potential influence of obesity on mortality beyond its established role in CVD.

## 2 Methods

We used National Health and Nutrition Examination Survey (NHANES) data from 1999 to 2004; NHANES is a series of surveys conducted by the Centers for Disease Control and Prevention (CDC) that assesses the U.S. population health and nutritional status through interviews and physical examinations. The surveys oversample participants from certain population subgroups, such as African Americans and Mexican Americans, to ensure the precision and reliability of subpopulation estimates. Detailed survey and laboratory methods are available on the NHANES website [13]. Conducted in 2‐year cycles with nationally representative samples, NHANES data equips researchers and policymakers with information to recognize health trends, design effective programs, and address health disparities for all Americans.

We included participants who were free of coronary artery disease or congestive heart failure, were older than 18 years, had BMI and NT‐proBNP levels measured, and whose mortality status was known. Demographic information, co‐morbid conditions, education levels, family income, smoking, and alcohol intake were self‐reported by participants using a computer‐assisted personal interview system. Physical examinations and laboratory sample collections were conducted in a mobile examination center by trained professionals. All participants provided informed consent and the National Center for Health Statistics Research Ethics Review Board approved all protocols.

We categorized age as young (< 45 years), middle‐aged (45–65 years), and elderly (> 65 years). Alcohol use was categorized as nondrinkers (< 12 drinks in a lifetime), light drinkers (< 5 drinks for men and < 4 drinks for women), and heavy drinkers (≥ 5 drinks for men and ≥ 4 drinks for women), adhering to the guidelines set by the US Department of Health and Human Services [14]. Smoking status categories were “current smokers,” defined as those currently smoking, “former smokers,” defined as individuals who smoked > 100 cigarettes in their lifetime, and “nonsmokers,” were those who never smoked or smoked 100 or fewer cigarettes in their lifetime. Participants reported the total family income for the preceding calendar year in dollars. Family income to poverty threshold ratio (FIPR) was calculated by dividing family income by the poverty guidelines for that year, as determined by The Department of Health and Human Services (HHS). FIPR was then categorized into five groups: 0–1, > 1–2, > 2–3, > 3–4, and > 4. Self‐reported education attainment was classified into less than high school education, high school graduate, some college education, and college graduate.

BMI was calculated by dividing weight (in kilograms) by height (in meters squared) [15]. We categorized individuals as normal weight if BMI < 25 kg/m^2^, overweight if BMI between 25 and < 30, and obese if BMI ≥ 30 [16]. Participants were categorized as hypertensives if their systolic blood pressure was > 140 mmHg and diastolic blood pressure was > 90 mm Hg, if they had a self‐reported history of hypertension, or if they were taking antihypertensive medications. We inferred the presence of diabetes mellitus if participants had a prior diagnosis of diabetes mellitus, were taking insulin or other oral medication for blood glucose management, had hemoglobin A1C > 7.0%, or had a blood glucose level > 200 mg/dL. Serum glucose was measured using Beckman Synchron LX20 (Beckman Coulter, Brea, Calif) and hemoglobin A1C was measured using the Primus CLC300 (Primus Corporation, Kansas City, MO). The cholesterol level was measured enzymatically in a series of coupled reactions that hydrolyze cholesteryl esters and oxidize the 3‐OH group of cholesterol. Serum creatinine levels were measured using the Jaffe rate method (kinetic alkaline picrate). We estimated glomerular filtration rate (GFR) using the Chronic Kidney Disease Epidemiology Collaboration equation [17].

NT‐proBNP measurements on stored samples were conducted using an electrochemiluminescence immunoassay (Roche Diagnostics Corp) on a Cobas e601 analyzer (Elecsys; Roche Diagnostics). The assay demonstrated a coefficient of variation ranging between 2.7% and 3.1%. Upper and lower detection limits for NT‐proBNP were established at 35 000 and 5 pg/mL, respectively. Values falling below the detection limit were adjusted and recoded to the detection limit divided by square root of 2, whereas values exceeding the upper detection limit were recoded to the predefined upper limit. We categorized NT‐proBNP into normal levels (< 126 pg/mL) or elevated (≥ 126 pg/mL) based on guidelines for asymptomatic individuals [5].

### 2.1 Outcome Ascertainment

Mortality data were obtained by probabilistically matching NHANES participants with death certificates from the National Death Index (NDI). Participants who lacked an NDI match were presumed alive. We categorized causes of death as cardiovascular (underlying cause due to heart disease), cancer (underlying cause due to malignant neoplasms), or other causes. NDI provided follow‐up information from survey participation to December 31, 2019. Only participants with sufficient identifying NDI matching data were eligible for mortality follow‐up. Follow‐up time was calculated as the difference between the NHANES examination date and the date of death, and participants alive as of December 31, 2019, were censored [18].

### 2.2 Statistical Methods

All analyses were performed using appropriate adjustments to survey weights for the three NHANES survey cycles. In NHANES, primary sampling units (used to estimate sampling error) represent variance units. These sampling weights were assigned to each person, reflecting adjustment for the unequal probability of selection, nonresponse, or noncoverage. Masked variance units are used to protect data confidentiality.

Descriptive statistics were calculated using mean with standard deviation, median with interquartile range, or frequencies, as appropriate. We calculated incidence rates and obtained raw estimates of differences in survival using Kaplan‐Meier survival curves and log‐rank tests. We used unadjusted and adjusted Cox proportional hazard models to explore the association between NT‐proBNP and all‐cause and cause‐specific mortality. Adjusted Cox models included age, gender, race, diabetes, hypertension, smoking, alcohol use, FIPR, education attainment, BMI categories, estimated GFR, and serum total cholesterol. We examined unadjusted and adjusted effect modification by BMI categories using interaction terms in statistical models for all‐cause and cause‐specific mortality. We also conducted two sensitivity analyses. First, we used unadjusted and adjusted competing risk models for cause‐specific mortality. Second, we examined if removing participants with < 1‐year follow‐up influenced the results.

## 3 Results

Of the 12 621 study participants, 6661 (52.8%) were females, 2416 (19.1%) were African Americans, 2650 (21.0%) were older than 65 years, 2466 (19.5%) were smokers, 3819 (30.3%) had hypertension, 966 (7.6%) had diabetes, and 3915 (31%) were obese. The mean (SD) age of the participants was 45.5 (19.9) years, BMI was 28.0 (6.2) kg/m^2^, and estimated GFR was 97.8 (24.8) mL/min. Median (IQR) of NT‐proBNP was 43.1 (72.2) pg/mL and 2256 (17.9%) participants had NT‐proBNP ≥ 126 pg/mL. The mean follow‐up was 16.1 (4.3) years and ranged from 0 to 20.7 years.

During 202 859 person‐years of follow‐up, 2794 (22.1%) participants died of which 816 (6.5%) were due to CVDs, 628 (5.0%) were due to cancer, and 1350 (10.7%) were due to other causes. Median survival for participants with higher NT‐proBNP ≥ 126 pg/mL was 13.7 (95%CI = 13.2, 14.4) years. However, median survival for participants with NT‐proBNP < 126 pg/mL could not be determined as more than half of the participants were still alive at the end of follow‐up. The mortality rate in participants with higher NT‐proBNP ≥ 126 pg/mL was 50.4 per 1000 person‐year as compared to 8.0 per 1000 person‐year in those with NT‐proBNP < 126 pg/mL. As expected, those who died were more likely to be older, males, hypertensives, diabetics, had high cholesterol, less than high school education, or had shorter follow‐up (Table 1).

**Table 1:** Study population characteristics by mortality status. Data are presented as mean (standard deviation) unless noted otherwise.

Variable	Alive (*N* = 9827)	Dead (*N* = 2794)	*p*‐value^a^
NT‐proBNP, pg/mL, median (IQR)	34.7 (49)	123.9 (232.0)	< 0.0001
NT‐proBNP ≥ 126 pg/mL, *N* (%)	872 (8.9)	1384 (49.5)	< 0.0001
Age, years	39.1 (16.1)	67.9 (14.9)	< 0.0001
< 45 years, *N* (%)	6450 (65.6)	254 (9.1)	< 0.0001
45–65 years, *N* (%)	2587 (26.3)	680 (24.3)	
> 65 years, *N* (%)	790 (8.0%)	1860 (66.6)	
Female, *N* (%)	5368 (54.6)	1293 (46.3)	< 0.0001
Hypertension, *N* (%)	2051 (20.9)	1768 (63.3)	< 0.0001
Diabetes Mellitus, *N* (%)	456 (4.6%)	510 (18.2%)	< 0.0001
Race			
White, *N* (%)	4426 (45.0)	1631 (58.4)	< 0.0001
Black, *N* (%)	1925 (19.6)	491 (17.6)	
Others, *N* (%)	3476 (35.4)	672 (24.0)	
Alcohol Use			
Nondrinkers, *N* (%)	1728 (17.6)	812 (29.1)	< 0.0001
Light drinkers, *N* (%)	5091 (51.8)	1647 (58.9)	
Heavy drinkers, *N* (%)	1112 (11.3)	193 (6.9)	
Smoking			
Nonsmokers, *N* (%)	4691 (47.7)	1148 (41.1)	< 0.0001
Past smokers, *N* (%)	1864 (19.0)	1035 (37.0)	
Current smokers, *N* (%)	1891 (19.2)	575 (20.6)	
Estimated GFR, mL/min	104.1 (21.5)	75.9 (23.1)	< 0.0001
BMI, Kg/m^2^	28.0 (6.3)	28.0 (5.9)	0.08
Normal weight (BMI < 25), *N* (%)	3,461 (35.2)	886 (31.7)	0.001
Overweight (BMI ≥ 25 to < 30), *N* (%)	3323 (33.8)	1036 (37.1)	
Obesity (BMI ≥ 30), *N* (%)	3043 (31.0)	872 (31.2)	
Cholesterol, mg/dL	198.0 (42.9)	208.5 (43.3)	< 0.0001
Family Income to Poverty Ratio	2.62 (1.66)	2.37 (1.48)	< 0.0001
0–1, *N* (%)	1914 (19.5)	483 (17.3)	< 0.0001
> 1–2, *N* (%)	2163 (22.0)	808 (28.9)	
> 2–3, *N* (%)	1363 (13.9)	455 (16.3)	
> 3–4, *N* (%)	1128 (11.5)	293 (10.5)	
> 4, *N* (%)	2560 (26.0)	480 (17.2)	
Follow‐up, years	17.8 (1.65)	9.9 (5.1)	< 0.0001
Education			
<High School, *N* (%)	2312 (23.5)	1147 (41.0)	< 0.0001
High School, *N* (%)	2020 (20.6)	673 (24.1)	
Some College, *N* (%)	2336 (23.8)	559 (20.0)	
College Graduate, *N* (%)	1782 (18.1)	374 (13.4)	

Abbreviations: BMI = body mass index, GFR = glomerular filtration rate, IQR = interquartile range, NT‐proBNP = N‐terminal pro‐brain natriuretic peptide.

^a^ Differences between the two groups were determined using Student's *t*‐test, Mann–Whitney test, or chi‐square test as appropriate.

### 3.1 All‐Cause Mortality, NT‐proBNP, and Effect Modification by Obesity

In the whole cohort, elevated NT‐proBNP ( ≥ 126 pg/mL) was significantly associated with all‐cause mortality in unadjusted (HR = 6.17; 95%CI: 5.53, 6.88; *p* < 0.001) and adjusted (HR = 1.82; 95%CI: 1.64, 2.03; *p* < 0.001) analyses than normal NT‐proBNP ( < 126 pg/mL). In normal‐weight individuals, unadjusted Cox regression models with interaction terms found that individuals with elevated NT‐proBNP levels had significantly higher risk of mortality than those with lower NT‐proBNP levels (HR = 7.48; 95%CI: 6.22, 9.01; *p* < 0.001). In obese individuals, elevated NT‐proBNP was associated with somewhat lower but still high risk of mortality than those with normal NT‐proBNP (HR = 7.08; 95%CI = 5.92, 8.47; *p* < 0.001); the difference in normal‐weight and obese mortality risks was significant (HR interaction: 0.64; 95%CI: 0.51, 0.81; *p* < 0.001).

**Figure 1:**
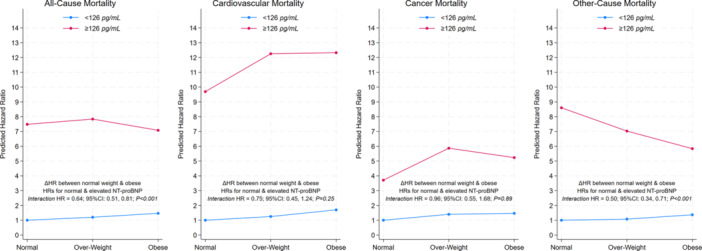
Effect modification by weight categories of the relationship between NT‐proBNP and all‐cause mortality and cause‐specific mortality. Survey‐weighted unadjusted Cox proportional hazard model comparing low (< 126 pg/mL) and high (≥ 126 pg/mL) NT‐proBNP levels. Weight categories were normal weight (BMI < 25 kg/m^2^) overweight (25 to < 30 kg/m^2^), and obesity (≥ 30 kg/m^2^). In the absence of obesity paradox, we would expect to see both lines parallel and the difference between the two lines to be not statistically significant.

After adjusting for potential confounders, similar results were noted (Figure 1). In normal‐weight and obese individuals, elevated NT‐proBNP was associated with significantly higher mortality risk than normal NT‐proBNP (HR = 2.05; 95%CI = 1.74, 2.41; *p* < 0.001 and HR = 1.32; 95%CI = 1.10, 1.58; *p* = 0.003, respectively); the difference between the normal‐weight and obese was significant (HR interaction =0.73; 95%CI = 0.59, 0.92; *p* = 0.008).

### 3.2 Cardiovascular Mortality, NT‐proBNP, and Effect Modification by Obesity

Elevated NT‐proBNP ( ≥ 126 pg/mL) showed a significant association with cardiovascular mortality compared to normal NT‐proBNP ( < 126 pg/mL) in both unadjusted (HR = 8.76; 95% CI: 7.17, 10.71; *p* < 0.001) and adjusted (HR = 2.34; 95% CI: 1.88, 2.91; *p* < 0.001) analyses. In unadjusted Cox regression models with an interaction term, normal‐weight and obese individuals with elevated NT‐proBNP levels exhibited a significantly increased risk of cardiovascular mortality compared to those with normal NT‐proBNP levels (HR = 9.69; 95%CI: 6.50, 14.44; *p* < 0.001 and HR = 12.3; 95%CI = 8.99, 16.9; *p* < 0.001, respectively). However, the difference in NT‐proBNP‐associated cardiovascular mortality risk between normal‐weight and obese individuals was not statistically significant (interaction HR: 0.75; 95%CI: 0.45, 1.24; *p* = 0.25). After adjusting for potential confounders, the difference in cardiovascular mortality remained nonsignificant. As compared to normal levels, elevated NT‐proBNP levels were associated with higher cardiovascular mortality risk in normal‐weight (HR = 2.31; 95% CI: 1.62, 3.30; *p* < 0.001) and obese individuals (HR = 1.93; 95%CI = 1.29, 2.90; *p* = 0.002) and there was no difference between the two groups of individuals (interaction HR: 0.91; 95%CI: 0.57, 1.45; *p* = 0.69) (Figure 1). These findings remained consistent when employing competing risks models (see Table S1) or after excluding participants with < 1‐year follow‐up (Table S2).

### 3.3 Cancer Mortality, NT‐proBNP, and Effect Modification by Obesity

In both adjusted and unadjusted analysis, elevated NT‐proBNP was significantly associated with cancer‐specific mortality as compared to normal NT‐proBNP in the whole cohort (HR = 3.83; 95%CI: 2.91, 5.04; *p* < 0.001 and HR = 1.50; 95%CI = 1.11, 2.03; *p* = 0.009, respectively).

In unadjusted Cox regression models with an interaction term, elevated NT‐proBNP levels were associated with significantly increased risk of cancer mortality than those with normal NT‐proBNP in normal‐weight (HR = 3.71; 95%CI: 2.29, 5.99; *p* < 0.001) and in obese individuals (HR = 5.23; 95%CI = 3.56, 7.68; *p* < 0.001). However, the difference in the NT‐proBNP‐associated mortality risks between the normal‐weight and obese individuals was not significant (interaction HR: 0.96; 95% CI: 0.55, 1.68; *p* = 0.89).

After adjusting for potential confounders, cancer‐specific mortality risk in elevated versus normal NT‐proBNP levels in normal‐weight individuals was no longer significant (HR = 1.28; 95%CI = 0.81, 2.02; *p* = 0.28) but remained significant in obese individuals (HR = 1.62, 95%CI = 1.08, 2.46; *p* = 0.02). Further, mortality risks between normal‐weight and obese were also similar (interaction HR: 1.21; 95% CI: 0.69, 2.14; *p* = 0.49) (Figure 1). These findings did not change after using competing risk models (see Table S1) or after excluding participants with < 1‐year follow‐up (Table S2).

### 3.4 Other‐Cause Mortality, NT‐proBNP, and Effect Modification by Obesity

Like all‐cause and cardiovascular and cancer mortality, elevated NT‐proBNP was associated with significantly higher other‐cause mortality in the whole cohort in both unadjusted (HR = 6.34; 95% CI: 5.53, 7.26; *p* < 0.001) and adjusted (HR = 1.74; 95% CI: 1.53, 1.98; *p* < 0.001).

In unadjusted Cox regression models with an interaction term and when comparing elevated with normal NT‐proBNP levels, normal‐weight and obese individuals had significantly increased risk of other‐cause mortality (HR = 8.60; 95% CI: 6.90, 10.73; *p* < 0.001 and HR = 5.84; 95%CI = 4.52, 7.53; *p* < 0.001, respectively); the difference in mortality risk between the normal‐weight and obese individuals was statistically significant (interaction HR: 0.50; 95% CI: 0.34, 0.71; *p* < 0.001).

After adjusting for potential confounders, and in contrast to cancer‐specific mortality, the other‐cause mortality risk between elevated and normal NT‐proBNP remained significant in normal‐weight individuals (HR = 2.27; 95%CI = 1.81, 2.85; *p* < 0.001) but was no longer significant for obese individuals (HR = 0.93, 95%CI = 0.73, 1.18; *p* = 0.55); however, the difference in NT‐proBNP‐associated other‐cause mortality risks between normal‐weight and obese individuals remained significant (interaction HR = 0.52; 95%CI = 0.36, 0.77; *p *= 0.001) (Figure 1). The results remained similar in competing risk models (see Table S1) or excluding participants with < 1‐year follow‐up (Table S2).

## 4 Discussion

In this study, we found that elevated NT‐proBNP in obese individuals free of CVD history demonstrated significantly lower all‐cause and other‐cause mortality rates compared to normal‐weight individuals with elevated NT‐proBNP. This finding was independent of potential confounders and provides evidence supporting the obesity paradox. We further confirm the previously reported association of elevated NT‐proBNP with all‐cause and cause‐specific mortality. However, we found no evidence to support the obesity paradox for cardiovascular or cancer mortality, as obesity did not modify the relationship between elevated NT‐proBNP and cardiovascular or cancer mortality. These results were robust and remained significant when competing causes of mortality were accounted for in the statistical analyses. These findings contribute to the existing literature on the obesity paradox and enhance the current understanding of the role of obesity in the relationship between NT‐proBNP and mortality in a population free from CVD.

The finding that obesity reduced mortality risk in individuals with elevated NT‐proBNP but without CVD expands the scope of the obesity paradox beyond those with a history of CVD. Obesity is known to be inversely related to NT‐proBNP levels; in heart failure patients, obese individuals tend to have lower NT‐proBNP levels compared to their nonobese counterparts. As a result, using established NT‐proBNP cutoffs in obese individuals is unlikely to significantly influence the well‐known inverse relationship between obesity and cardiovascular mortality (the obesity paradox) [19]. However, the absence of an attenuating effect of obesity on cardiovascular mortality in individuals with elevated NT‐proBNP was unexpected. Elevated NT‐proBNP levels may indicate subclinical or undiagnosed heart failure, with prior studies noting obesity's attenuation of mortality risk in this population [2, 3]. Our findings indicate that accounting for NT‐proBNP levels may nullify the protective effect of obesity on cardiovascular mortality. A notable finding is that obesity diminishes the risk of other‐cause (noncardiovascular and noncancer) mortality in individuals with elevated NT‐proBNP. This effect is unlikely to be attributable to the obesity paradox in subclinical heart failure, as we observed no rise in cardiovascular mortality. Instead, it may stem from obesity's protective influence in other disease contexts as epidemiological studies have suggested a protective role of obesity in noncardiovascular conditions such as COPD and pulmonary hypertension [20, 21].

Obesity's impact on various diseases, including cardiovascular conditions, is intricate and influenced by factors such as adipose tissue distribution, adipokine release, and type of adipose tissue expansion [22]. The mechanisms underlying the obesity paradox remain elusive. One proposed mechanism, the endotoxin‐lipoprotein hypothesis, posits that elevated lipid levels in obese individuals may neutralize circulating endotoxins, potentially mitigating inflammation in the heart [23]. Another explanation links the obesity paradox to heart failure being a catabolic disease, suggesting that obese individuals possess greater metabolic capacity and experience less muscle wasting, leading to improved prognoses compared to those with healthy weights [24]. Furthermore, enhanced stimulation of the renin‐angiotensin‐aldosterone system is posited as a contributing factor, with overweight individuals demonstrating better tolerance to antihypertensive drugs, potentially resulting in improved outcomes [25]. Recent research suggests a combined effect of obesity, high‐fat diet, and disrupted circadian rhythm on the enhancement of lipid metabolic genes and cardiac function [26]. Some argue that individuals with normal BMI are often perceived as healthy, leading to missed or delayed diagnoses of underlying health issues and negatively impacting overall health and survival [27]. Lastly, some suggest that the obesity paradox may stem from biases associated with the limitations of anthropometric indices in classifying obesity [22].

The study's findings highlight a broader presence of the obesity paradox, potentially complicating public health recommendations, as weight reduction interventions may not universally benefit all individuals, particularly in older adults or those with specific health conditions. However, it emphasizes the necessity for personalized public health strategies, considering factors beyond BMI, such as body composition and metabolic health that may be more prudent. While the complexity of the obesity paradox warrants further investigation, efforts to promote healthy lifestyles and manage weight should continue. Clinical research should explore the mechanisms behind the paradox, including adipose tissue types and genetic variations, aiming to develop novel therapeutic strategies. Effective communication of the nuances of the obesity paradox by healthcare professionals is crucial, alongside the development of improved risk stratification methods within the obese population. The implications of the obesity paradox vary globally, requiring culturally sensitive approaches to obesity prevention and management.

This study has notable strengths and potential limitations. A primary strength lies in its substantial sample size and comprehensive analysis, which incorporates various confounding variables. The considerable number of participants enhances the statistical power of the analyses, facilitating robust sensitivity analyses. Additionally, the extended mean follow‐up period of 16 years enables thorough exploration into the enduring impacts of BMI on the relationship between NT‐proBNP and mortality outcomes. Hence, there is a critical need for future investigations to delve into adipocyte gene expression and its potential influence on decreased mortality risk.

In conclusion, we report the presence of an obesity paradox in individuals free of CVD by showing that obese individuals with elevated NT‐proBNP levels had lower all‐cause and other‐cause mortality rates compared to normal‐weight individuals with elevated NT‐proBNP, supporting the obesity paradox. The obesity paradox was not present for cardiovascular or cancer mortality outcomes. The underlying mechanisms need further exploration but may include complex interactions between adipose tissue and other organs.

## Supporting information

Supporting information.

## Data Availability

The data that support the findings of this study are available in NHANES at https://wwwn.cdc.gov/nchs/nhanes/Default.aspx. These data were derived from the following resources available in the public domain: NHANES, https://wwwn.cdc.gov/nchs/nhanes/Default.aspx. Data is available publicly and references are cited within manuscript.
