# Supplementary material for: Obesity Paradox and the Effect of NT‐proBNP on All‐Cause and Cause‐Specific Mortality
Source: Clin Cardiol. 2024 Nov 8;47(11):e70044. doi: 10.1002/clc.70044 (PMC11546245; doi:10.1002/clc.70044)
Supplement: Supplementary file 1 — Supporting information. [file CLC-47-e70044-s001.docx]

**Supplementary Materials**

**Title:** Obesity Paradox and the Effect of NT-proBNP on All-cause and Cause-specific Mortality

**Abbreviated Title:** Obesity paradox, NT-proBNP and Mortality

**Authors:** Rupinder Kaur Bahniwal, MBBS; Nargiza Sadr, BSN; Colleen Schinderle, BS; Cynthia J. Avila, MBS; Julie Sill, Ph.D., M.DEHS; Rehan Qayyum, MD, MHS

**Supplementary Table 1:** Results of unadjusted and adjusted competing risk models.

| **Variable** | **Cardiovascular Mortality** | **Cancer Mortality** | **Other Mortality** |
| --- | --- | --- | --- |
| **Unadjusted** | | | |
| ***NT-proBNP levels*** | | | |
| NT-proBNP <126 | Reference (1) | Reference (1) | Reference (1) |
| NT-proBNP >125 | 7.52 (5.01, 11.27); <0.001 | 2.88 (1.99, 4.16); <0.001 | 7.32 (5.79, 9.26); <0.001 |
| ***Body Mass Index Categories*** | | | |
| Normal | Reference (1) | Reference (1) | Reference (1) |
| Overweight | 1.24 (0.83, 1.85); 0.29 | 1.40 (1.02, 1.92); 0.04 | 1.07 (0.84, 1.35); 0.58 |
| Obese | 1.67 (1.14, 2.45); 0.009 | 1.45 (1.01, 2.08); 0.04 | 1.35 (1.04, 1.75); 0.03 |
| ***Interaction between NT-proBNP levels and Body Mass Index*** | | | |
| NT-proBNP>125 x Overweight | 1.04 (0.62, 1.73); 0.89 | 1.15 (0.74, 1.78); 0.54 | 0.74 (0.56, 0.98); 0.04 |
| NT-proBNP>125 x Obese | 0.81 (0.52, 1.24); 0.34 | 1.02 (0.69, 1.49); 0.92 | 0.49 (0.36, 0.67); <0.001 |
| **Adjusted** | | | |
| ***NT-proBNP levels*** | | | |
| NT-proBNP <126 | Reference (1) | Reference (1) | Reference (1) |
| NT-proBNP >125 | 1.73 (1.13, 2.64); 0.01 | 0.98 (0.66, 1.46); 0.91 | 1.93 (1.48, 2.53); <0.001 |
| ***Body Mass Index Categories*** | | | |
| Normal | Reference (1) | Reference (1) | Reference (1) |
| Overweight | 0.80 (0.53, 1.22); 0.30 | 1.02 (0.74, 1.40); 0.91 | 0.74 (0.60, 0.93); 0.01 |
| Obese | 1.04 (0.69, 1.57); 0.84 | 1.16 (0.81, 1.67); 0.42 | 0.92 (0.73, 1.16); 0.48 |
| ***Interaction between NT-proBNP levels and Body Mass Index*** | | | |
| NT-proBNP>125 x Overweight | 1.26 (0.76, 2.09); 0.37 | 1.37 (0.86, 2.17); 0.19 | 0.84 (0.62, 1.12); 0.24 |
| NT-proBNP>125 x Obese | 1.04 (0.69, 1.56); 0.86 | 1.36 (0.87, 2.14); 0.18 | 0.52 (0.38, 0.71); <0.001 |
| ***Footnote:*** *Adjusted models include the following variables: age, gender, race/ethnicity, body mass index, hypertension, diabetes, smoking, alcohol use, cholesterol levels, serum c-reactive protein, estimated glomerular filtration rate, education level, and family income to poverty threshold ratio.* | | | |

**Supplementary Table 2:** Results of unadjusted and adjusted Cox proportional Hazard models after excluding individuals who died during the first year of follow-up.

| **Variable** | **Cardiovascular Mortality** | **Cancer Mortality** | **Other Mortality** |
| --- | --- | --- | --- |
| **Unadjusted** | | | |
| ***NT-proBNP levels*** | | | |
| NT-proBNP <126 | Reference (1) | Reference (1) | Reference (1) |
| NT-proBNP >125 | 10.2 (6.76, 15.4); <0.001 | 3.33 (1.99, 5.58); <0.001 | 8.59 (6.86, 10.8); <0.001 |
| ***Body Mass Index Categories*** | | | |
| Normal | Reference (1) | Reference (1) | Reference (1) |
| Overweight | 1.35 (1.00, 1.82); 0.05 | 1.44 (1.01, 2.06); 0.04 | 1.07 (0.83, 1.38); 0.58 |
| Obese | 1.80 (1.33, 2.45); <0.001 | 1.52 (1.11, 2.08); 0.009 | 1.34 (1.06, 1.70); 0.02 |
| ***Interaction between NT-proBNP levels and Body Mass Index*** | | | |
| NT-proBNP>125 x Overweight | 0.94 (0.60, 1.49); 0.80 | 1.27 (0.75, 2.17); 0.36 | 0.75 (0.57, 1.00); 0.05 |
| NT-proBNP>125 x Obese | 0.71 (0.42, 1.20); 0.20 | 1.03 (0.56, 1.89); 0.92 | 0.49 (0.34, 0.71); <0.001 |
| **Adjusted** | | | |
| ***NT-proBNP levels*** | | | |
| NT-proBNP <126 | Reference (1) | Reference (1) | Reference (1) |
| NT-proBNP >125 | 2.43 (1.67, 3.53); <0.001 | 1.17 (0.72, 1.90); 0.52 | 2.28 (1.81, 2.87); <0.001 |
| ***Body Mass Index Categories*** | | | |
| Normal | Reference (1) | Reference (1) | Reference (1) |
| Overweight | 0.77 (0.54, 1.09); 0.14 | 0.96 (0.66, 1.39); 0.84 | 0.67 (0.53, 0.85); 0.001 |
| Obese | 0.97 (0.67, 1.38); 0.85 | 1.11 (0.76, 1.62); 0.59 | 0.77 (0.61, 0.98); 0.03 |
| ***Interaction between NT-proBNP levels and Body Mass Index*** | | | |
| NT-proBNP>125 x Overweight | 1.05 (0.68, 1.63); 0.81 | 1.43 (0.86, 2.38); 0.16 | 0.82 (0.62, 1.08); 0.16 |
| NT-proBNP>125 x Obese | 0.86 (0.52, 1.42); 0.55 | 1.29 (0.70, 2.38); 0.41 | 0.52 (0.36, 0.76); 0.001 |
| ***Footnote:*** *Adjusted models include the following variables: age, gender, race/ethnicity, body mass index, hypertension, diabetes, smoking, alcohol use, cholesterol levels, serum c-reactive protein, estimated glomerular filtration rate, education level, and family income to poverty threshold ratio.* | | | |
